# Evaluation of primary HPV-DNA testing in relation to visual inspection methods for cervical cancer screening in rural China: an epidemiologic and cost-effectiveness modelling study

**DOI:** 10.1186/1471-2407-11-239

**Published:** 2011-06-13

**Authors:** Ju-Fang Shi, Karen Canfell, Jie-Bin Lew, Fang-Hui Zhao, Rosa Legood, Yan Ning, Leonardo Simonella, Li Ma, Yoon-Jung Kang, Yong-Zhen Zhang, Megan A Smith, Jun-Feng Chen, Xiang-Xian Feng, You-Lin Qiao

**Affiliations:** 1 Department of Cancer Epidemiology, Cancer Institute, Chinese Academy of Medical Sciences, Peking Union Medical College, 17, South Panjiayuan LN, PO Box 2258, Beijing 100021, China; 2 Cancer Epidemiology Research Unit, Cancer Council NSW, 153 Dowling Street, Woolloomooloo 2011, New South Wales, Australia; 3 School of Public Health, University of Sydney, Australia; 4 The London School of Hygiene and Tropical Medicine, London, WC1E 7HT, United Kingdom; 5 School of Public Health, Dalian Medical University, Dalian 116044, Liaoning Province, China; 6 Shanxi Cancer Institute/Hospital, Taiyuan 030013, Shanxi Province, China; 7 Changzhi Medical College, Changzhi 046000, Shanxi Province, China

## Abstract

**Background:**

A new lower-cost rapid-throughput human papillomavirus (HPV) test (*care*HPV, Qiagen, Gaithersburg, USA) has been shown to have high sensitivity for the detection of high grade cervical intraepithelial neoplasia.

**Methods:**

We assessed the outcomes and cost-effectiveness of *care*HPV screening in rural China, compared to visual inspection with acetic acid, when used alone (VIA) or in combination with Lugol's iodine (VIA/VILI). Using data on sexual behaviour, test accuracy, diagnostic practices and costs from studies performed in rural China, we estimated the cost-effectiveness ratio (CER) and associated lifetime outcomes for once-lifetime and twice-lifetime screening strategies, and for routine screening at 5-yearly, 10-yearly and IARC-recommended intervals. The optimal age range for once-lifetime screening was also assessed.

**Results:**

For all strategies, the relative ordering of test technologies in reducing cervical cancer incidence and mortality was VIA (least effective); VIA/VILI; *care*HPV@1.0 pg/ml and *care*HPV@0.5 pg/ml (most effective). For once-lifetime strategies, maximum effectiveness was achieved if screening occurred between 35-50 years. Assuming a participation rate of ~70%, once-lifetime screening at age 35 years would reduce cancer mortality by 8% (for VIA) to 12% (for *care*HPV@0.5) over the long term, with a CER of US$557 (for VIA) to $959 (for *care*HPV@1.0) per life year saved (LYS) compared to no intervention; referenced to a 2008 GDP per capita in Shanxi Province of $2,975. Correspondingly, regular screening with an age-standardised participation rate of 62% (which has been shown to be achievable in this setting) would reduce cervical cancer mortality by 19-28% (for 10-yearly screening) to 43-54% (using IARC-recommended intervals), with corresponding CERs ranging from $665 (for 10-yearly VIA) to $2,269 (for IARC-recommended intervals using *care*HPV@1.0) per LYS.

**Conclusions:**

This modelled analysis suggests that primary *care*HPV screening compares favourably to visual inspection screening methodologies in rural China, particularly if used as part of a regular screening program.

## Background

Cancer of the uterine cervix is associated with a substantial burden of disease and is a significant cause of death amongst women in the People's Republic of China. The number of cases of cervical cancer in China is estimated to have increased by 14% over the period from 2000 to 2005 [1]. In the absence of substantial intervention, the number of new cases is projected to further increase over the next several decades, due to population ageing [2]. There may be considerable heterogeneity in rates of cervical cancer within China, and the evidence is consistent with a higher burden of disease in some rural areas [2].

Various approaches to screening in rural China and other low resource settings have been considered. Given the critical nature of the role of human papillomavirus (HPV) infection in the causation of cervical cancer, a potential screening strategy is to perform primary testing for infection with high risk types of HPV. However, to date, the higher cost of HPV DNA testing has precluded its use as part of large scale screening programs in low resource settings. More recently, efforts have been made to develop a lower cost rapid throughput test. *Care*HPV is a new technology which has been developed via a public/private partnership between Qiagen Inc (Gaithersburg, MD) and PATH (Seattle, WA, USA), which has been shown to have high sensitivity for detection of cervical intraepithelial neoplasia grade 2 and above (CIN2+) in rural China [3]. The only realistic currently available screening alternatives to *care*HPV in this setting are visual inspection techniques; cytology-based approaches are difficult to implement due to the need for high levels of quality assurance and ongoing training, and HPV screening with technologies used in developed countries, such as Hybrid Capture 2 (HC2, Qiagen, Gaithersberg, MD), is likely to be too expensive to implement on a large scale. HPV vaccination offers an additional cervical cancer prevention strategy, but if it should become available and affordable in rural China in the near future, optimal outcomes will be achieved if vaccination of younger cohorts is implemented in conjunction with screening of older cohorts [4].

The objective of the current study was to perform a comprehensive assessment of the cost-effectiveness and impact on cancer incidence and mortality of various screening strategies, focusing on assessing the relative benefits of *care*HPV and visual inspection screening in rural China. We utilised detailed epidemiological data on sexual behaviour, HPV infections, test accuracy and costs in rural Shanxi province [3,5,6] to construct a mathematical model of HPV infection, cervical intraepithelial neoplasia (CIN), and cervical screening. We simulated screening with *care*HPV at 0.5 pg/ml and 1.0 pg/ml thresholds, and compared the findings to those for screening with visual inspection using acetic acid, when used alone (VIA) or in combination with Lugol's iodine (VIA/VILI). For each screening test, a number of different strategies were considered, including once- or twice-lifetime mobile screening strategies which would screen women aged 35 and/or 45 years; or regular program-based screening among women aged 30-59 years, at 10-yearly, 5-yearly or International Agency for Research on Cancer (IARC)-recommended intervals (for cytology) of every 3 years in women aged 25-49 years and every 5 years in women aged 50-64 years [7]. We calculated the cost-effectiveness of each of these potential strategies, in order to provide data for health care decision makers on potentially feasible screening options using currently available technology.

## Methods

### Structure and parameterisation of the HPV transmission and natural history models

We performed a dynamic simulation of HPV transmission in rural China, which was based on a reanalysis of self-reported sexual behavioral data for a population of 662 females aged 15-59 years in Yangcheng County, Shanxi Province; these data were originally collected via a survey performed by IARC and the Cancer Institute of Chinese Academy of Medical Sciences (CICAMS) [5]. The structure of the transmission model was based on previously developed models for Finland and Australia [8,9]. No data on male or high risk female behaviour were available from the survey. Therefore, we assumed that male behaviour was as reported for females, and took a fitting approach to estimate behaviour in high risk groups; adjusting the annual number of the partnerships in a small group (9%) of males and females over 40 years of age, such that the predicted HPV prevalence in females corresponded to the observed age-specific cross-sectional prevalence in the IARC survey [5] (Table 1).

**Table 1:** Model parameters for screening, diagnosis, treatment and utilities; and ranges for sensitivity analysis


**Parameter**	**Baseline value**	**Range for sensitivity analysis**

**Screening and test characteristics**		
Screening participation rate for once-lifetime screening (%)†	71	40 - 100
Proportion of women never screened in lifetime in program-based screening (%)††	15	
Total participation rate over one screening round for program-based screening (age-standardised to local population for 30-59 years) (%)†	62	35 - 100
Rate of technically inadequate tests for *care*HPV (%)††	0	0 - 10
Rate of loss to follow-up after screening, diagnosis or treatment (if follow-up not performed the same day) (%)††	10	5 - 20
Age-specific rate of unsatisfactory visual tests if performed as first visual test in management process (VIA, VIA/VILI or colposcopy) (%)‡		
20-34 years	5	0 - 5
35-39 years	6	0 - 6
40-44 years	7	0 - 7
45-49 years	16	0 - 16
50-54 years	35	0 - 35
55+ years	59	0 - 59
Conditional probability of an unsatisfactory colposcopy, given a prior unsatisfactory visual test (%)‡	92	80 - 100
LEEP treatment success rate (%) [12]	93.6	90 - 100
Age-standardised annual progression rate from CIN3 to cancer (%) [11]	1.4	0.7 - 2.8
Proportion of CIN3 treated by hysterectomy in rural Chinese settings (%)	21.1	5 - 21.1
5-year survival by FIGO stage (%) [13] (+/-10% of baseline values)		
FIGO I	88.0	79.1 - 96.7
FIGO II	68.0	61.2 - 74.8
FIGO III	41.3	37.2 - 45.5
FIGO IV	15.5	13.9 - 17.0
**Sexual behaviour parameters**		
Per-partnership HPV transmission probability [9]	0.6	-
Age group and sexual behaviour group mixing probability††		
Same five-year age group mixing	0.7	-
Random age group mixing	0.3	-
Same sexual activity group mixing (four activity groups)	0.7	-
Random sexual activity group mixing	0.3	-
Average age-specific new annual partnerships (across sexual activity groups) in females/ males‡‡		
10-14 years	0.000/ 0.000	-
15-19 years	0.097/ 0.094	-
20-24 years	0.316/ 0.263	-
25-29 years	0.029/ 0.029	-
30-34 years	0.008/ 0.008	-
35-39 years	0.045/ 0.053	-
40-44 years	0.039/ 0.047	-
45-50 years	0.075/ 0.075	-
50-54 years	0.021/ 0.021	-
55+ years	0.011/ 0.011	-
Aggregated costs (in US$)*		
VIA ($) - once or twice-lifetime (mobile) screening at district hospital	3.55	2.84 - 4.26
VIA ($) - program-based screening at county hospital	4.30	3.44 - 5.16
VILI ($) - district or county hospital	0.40	0.32 - 0.48
*care*HPV total cost - self-sampling ($) (including labour cost, assuming test cost = US$5)	9.20	7.20 - 14.20
*care*HPV total cost - provider-sampling ($) (including labour cost, assuming test cost = US$5)	10.34	8.34 - 15.34
LEEP ($)	55.95	44.76 - 67.13
Cancer treatment cost ($):		
FIGO I	627.64	502.11 - 753.16
FIGO II	1953.20	1562.56 - 2343.83
FIGO III	1810.17	1448.13 - 2172.20
FIGO IV	662.61	530.09 - 795.1
Discount rate (%) [24]	3.6	0 - 5
**Secondary analysis of utilities (reference = 1**.**0 for perfect health) **[19-21]		
Having a screening test	0.999945	0.999616 - 0.999956
Screening test positive with no treatment on the same day	0.999918	0.998849 - 0.999934
Colposcopy negative	0.999877	0.998274 - 0.999901
Colposcopy positive and biopsy negative	0.9965	0.992603 - 0.997238
Colposcopy positive then biopsy confirmed CIN1 with no treatment	0.9965	0.992603 - 0.997238
Colposcopy positive then biopsy confirmed CIN1 with LEEP treatment	0.984	0.935178 - 0.987178
Colposcopy positive then biopsy confirmed CIN2-3 with LEEP treatment	0.984	0.935178 - 0.987178
Colposcopy positive then biopsy confirmed CIN3 with hysterectomy treatment	0.85	0.82 - 0.88
Cancer - FIGO I	0.76	0.65 - 0.76
Cancer - FIGO II	0.67	0.56 - 0.67
Cancer - FIGO III	0.67	0.56 - 0.67
Cancer - FIGO IV	0.67	0.48 - 0.67

†	 Data source: Professor You-Lin Qiao, personal communication (based on a demonstration screening project in Xiangyuan County, Shanxi Province 2006);

†† Assumption (no data available);

‡ Re-analysis of data on colposcopy unsatisfactory rates from Dai et al [5];

‡‡ Female data were based on re-analysis of the IARC/CICAMS study [5], and male sexual contact data were assumed based on female data;

* Micro-costing study results as described in text. Range for sensitivity analysis +/-20% of baseline values.

We adapted and updated a previously developed Markov model of CIN and invasive cervical cancer natural history [10], which was informed by published data on the probability of CIN3 progressing to invasive cervical cancer [11]. We used a standard assumption in cervical cancer evaluations, that CIN natural history (but not HPV infection) is similar in different populations worldwide. The parameters for CIN progression and regression (but not for HPV infection rates) were based on a review of the literature [12] and were then more precisely estimated, as previously described, using data from a well-characterised screened population (Australia) after accounting for detailed screening behaviour and screening test accuracy [12]. For rural China, stage-specific invasive cancer survival was based on average survival rates from the International Federation of Gynecology and Obstetrics (FIGO) [13]. In order to calculate age-standardised cancer incidence and mortality rates, we applied the world standard population according to Ahmad et al [14]. Because comprehensive local data on cervical cancer incidence and mortality were not available, we assessed the predicted age-specific cancer incidence in relation to the average value for 24 less developed countries using data from IARC's Cancer Incidence in Five Continents [15].

### Structure and parameterisation of the screening, diagnosis and treatment model

Shanxi province is divided into 119 counties incorporating over 1000 townships or communities, with villages being the lowest administrative units within each community. Villages are usually equipped with a small health clinic, whereas large hospitals are generally located in the county-level cities. The screening and management pathways considered in this study included: mobile screening conducted by screening teams travelling to villages, visiting each village on average either once or twice in the lifetime of the target population; versus programs for repeated recall for screening at centralised county hospitals, which would potentially occur at 10-yearly, 5-yearly or IARC-recommended intervals. These viable and locally acceptable methods of screening organisation were determined in consultation with local clinicians and CICAMS epidemiologists (Personal Communication, Professor You-Lin Qiao). Screening participation assumptions were informed by unpublished data from a government-sponsored VIA/VILI screening demonstration project in 3,492 women aged 30-59 years who were screened using VIA/VILI in Xiangyuan, Shanxi Province in 2006 (Personal Communication, Professor You-Lin Qiao). Based on these data, we assumed an overall participation rate of 71% for one screening round implemented by a mobile service. To take into account the reduced screening coverage reported at older ages in the demonstration project, for regular program-based screening conducted at county hospitals we assumed age-specific participation patterns, with an overall age-standardised participation rate of 62% in screened age groups (Table 1).

**Figure 1:**
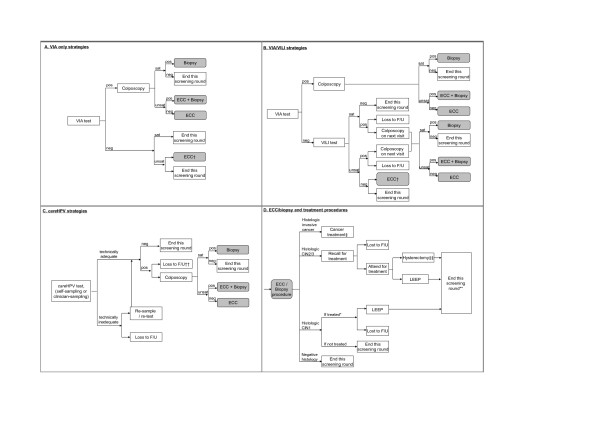
**Screening and management pathways**. pos = positive result; neg = negative result; sat = original cervical squamocolumnar junction fully visible. † A proportion (10%) of women with unsatisfactory and negative VIA or VILI test results were assumed to undergo endocervical curettage (ECC) on the same day. †† If colposcopy performed on the same day as HPV sampling and laboratory testing, assumed no loss to follow-up. ‡ Model assumes stage-specific cancer survival as described in text. ‡‡ A proportion (21%) of women with CIN3 were assumed to receive hysterectomy treatment, based on data from the micro-costing study.* CIN1 is assumed to be treated in once or twice-lifetime strategies only. ** Go to a sub-model of post-treatment natural history and recurrence [12].

Figure 1 depicts the structure of the screening, diagnosis and treatment pathways used for each of the test modalities; model parameters for screening and diagnosis are summarised in Tables 1 and 2. For VIA, test positives were assumed to have immediate colposcopy, and therefore no women were lost to diagnostic follow-up; for combined VIA/VILI strategies, VIA positives had immediate colposcopy whereas VIA negatives underwent VILI testing. However, VILI-positive women could not have immediate colposcopy since the use of Lugol's iodine precludes full colposcopic examination with acetic acid; in this case colposcopy after positive VILI was assumed to be performed on the next day with some women (10%) lost to follow-up. An age-specific probability of having unsatisfactory visual inspection results (in which the original squamocolumnar junction was not fully visible) was incorporated using data from a previous study [5]. A proportion (10%) of women with unsatisfactory but negative VIA or VILI test results were assumed to undergo endocervical curettage (ECC) on the same day (Figure 1). We also incorporated an age-specific unsatisfactory rate for colposcopy, which was based on local data [5,16].

**Table 2:** Summary of test characteristics for detection of CIN2+†


	**VIA only **	**VIA/VILI**	***care*HPV@ 1.0 pg/ml**	***care*HPV@ 0.5 pg/ml**	**Colposcopy**

Sensitivity	41%	58%	84% (provider) 73% (self)	90% (provider) 81% (self)	81%
Specificity	95%	82%	88% (provider) 88% (self)	84% (provider) 82% (self)	77%

† Note that this table summarises the finding for sensitivity and specificity in the original study populations, taking into account the relative proportions of CIN1, 2 and 3 in the population. However, the model implementation used these data to derive a test probability matrix separately describing the relationship between each health state (Normal; PCR HPV positive; CIN1, CIN2, CIN3+, etc.) and each test, rather than utilising the study sensitivity and specificity estimates directly, as described in the text [3,5,6].

For *care*HPV strategies, alternative sampling methods and different thresholds for test positivity were considered. In mobile screening strategies involving one or twice lifetime screening, it was assumed that women would perform self-sampling, and that specimen analyses would occur in a community or village clinic. For regular screening strategies where women attend the county hospital for screening, cervical samples were assumed to be taken by a health care provider. In both cases it was assumed that samples were processed on the same day, with HPV-positive women receiving immediate colposcopy and directed biopsy (as required).

For all strategies, we assumed diagnostic confirmation of the screening results would occur prior to any treatment, because after extensive consultation with local decision-makers it was determined that in this setting "see-and-treat" procedures would not currently be clinically acceptable. The diagnostic process involved colposcopy, directed biopsy, and ECC for a proportion of women with unsatisfactory colposcopy (Figure 1). The majority of women with a histologically-confirmed CIN2/3 were assumed to be treated with the loop electrosurgical excision procedure (LEEP). For once or twice lifetime screening strategies, we also assumed that all histologically-confirmed CIN1 were treated by LEEP. We applied a baseline estimate of a 94% success rate for LEEP based on a previous review [12] (evaluating the range 90%-100% in sensitivity analysis).

For each screening test, we characterised the probability of a positive test result given a satisfactory test and given the underlying health states in the natural history model, which included normal, HPV infected (approximated as PCR-HPV positive), CIN1, CIN2, CIN3 and stage-specific undiagnosed and diagnosed cancer states. In order to characterise the accuracy of *care*HPV testing, we used the findings of a cross-sectional study conducted in 2,388 women in rural Shanxi Province [3]. In this study, *care*HPV testing from cervical samples taken by a health provider was found to have sensitivities for CIN2+ of 90% and 84% at cut-off thresholds of 0.5 pg/ml and 1.0 pg/ml, respectively; with corresponding test specificities of 84% and 88%. The study also found that the sensitivity of *care*HPV testing for CIN2+ when women provided self-sampled vaginal specimens was comparable but slightly lower than that of cervical specimens (Table 2). Two studies conducted in Shanxi Province have examined the accuracy of VIA screening compared to a "gold standard" diagnosis based upon 4-quadrant biopsy; and both found that in this setting the sensitivity of VIA testing was low at 41-46%, but with a corresponding high specificity of 92-95% [3,17] and we used these data in the model (Table 2). To characterise VILI accuracy after negative VIA, we reanalysed data from a Yangcheng County study [5,16] and adjusted the sensitivity of VILI testing downwards to account for a potential inflation of up to 20 percentage points in sensitivity in studies of visual inspection which use colposcopic-directed biopsy as the gold standard [17]. For colposcopy performance, we used data from a study which assessed colposcopic accuracy against 4-quadrant biopsy, which found that colposcopy had an 81% sensitivity for CIN2+ [6]. The assumed test characteristics are summarised in Table 2.

### Costs

We performed a micro-costing study for costs associated with the delivery of screening and diagnostic tests and LEEP at the Women and Children's Hospital in Xiangyuan County, Shanxi Province in April 2008. This hospital has been the focus of previous population-based cervical screening studies and demonstration projects [18]. The costing study was conducted as one component of a larger government-sponsored cervical screening demonstration project, which was approved by the Institutional Review Board of the Cancer Foundation of China. The costing was undertaken from a societal perspective and included both direct medical costs and direct nonmedical costs. The resources (consumables, equipment and staff time) associated with clinic visits and laboratory testing were identified using observational field work and extensive consultation with local medical staff. Each item was costed at 2008 prices from financial records at the hospital. Transportation costs were estimated based on a prior demonstration project in Shanxi Province [18], in which the women were transferred between their home villages and the county hospital for screening (personal communication, Prof. You-Lin Qiao). We also conducted a detailed assessment of the overhead costs associated with delivering the screening program, including infrastructure, recruitment, data administration, training, quality control and staff transportation costs. For attribution of these overhead costs we estimated that on average 60 women would be screened each day after full implementation of the screening initiative. The test cost for *care*HPV in the base case was assumed to be US$5, not including labour and overhead costs.

Invasive cancer treatment costs by the FIGO stage were also obtained from the government-sponsored demonstration project in Shanxi Province. These direct medical costs were obtained from an audit of hospital charges in three hospitals: the Cancer Hospital of Shanxi Province (a provincial-level referral centre), the affiliated hospitals of Changzhi Medical College, Changzhi City (city level) and the Women and Children's Hospital of Xiangyuan County (county level). To identify the direct nonmedical costs associated with invasive cancer treatment, 192 patients with invasive cervical cancer were interviewed at the three hospitals. For the current analysis we assumed a charge/cost ratio of 1.25, and we also assumed that all FIGO I and FIGO IV patients are treated at county hospitals and that FIGO II/III patients are treated at city hospitals (50%) and provincial hospitals (50%). The final aggregated costs for screening, diagnosis and treatment procedures are presented in Table 1.

### Utilities

The primary results of the current analysis were based on estimates of life-years saved (LYS). However, as a secondary analysis, we also performed an indicative evaluation of the impact on quality-adjusted life years saved (QALYs) for various screening strategies. In order to estimate QALYs, some health states in the model were assigned a disutility value. These included utilities which reflected the experience of being screened; having a positive screening test; having a confirmed precancerous lesion with treatment; and having invasive cervical cancer (according to stage). The weightings were informed by an international literature review, because comprehensive information on utilities are not available for rural China (Table 1) [19-21].

### Effectiveness and resource utilisation outcomes

For each strategy we estimated the age-standardised incidence and mortality of invasive cervical cancer (across all ages) and the cumulative lifetime risk of developing invasive cancer to age 65 years. For once-lifetime screening strategies we also calculated the number of colposcopies, biopsies and treatments associated with the use of each screening test, and the ratio of the number of colposcopies to the number of treatments performed. Furthermore, for once-lifetime screening, we performed an analysis of the optimal age of screening by calculating the life years saved for a range of screening ages. Because this analysis was designed to assess the optimal age range for a single round of screening in a population, we did not discount the life years saved for the calculation.

### Cost-effectiveness analysis

We took a societal perspective for the cost-effectiveness analysis, in accordance with World Health Organization (WHO) recommendations for health economic assessments [22]. All costs and cost-effectiveness ratios were originally calculated in Chinese Yuan (CNY) and then converted to 2009 US dollars (exchange rate: 1 USD = 6.8304 CNY; 19 May 2009). All costs and effects were discounted (according to standard methods [23]) at 3.6% (the interest rate used by the Bank of China in 2008) [24]. The 3% discount rate recommended by WHO for cost-effectiveness assessments in developing countries [22] was included in the range assessed in sensitivity analysis. Cost-effectiveness ratios (CERs) were compared to an indicative threshold value of the estimated gross domestic product (GDP) per capita in Shanxi Province in 2008 [25], which was equivalent to US$2,975. Strategies less than this value are considered "very cost-effective"; and all strategies less than three times this value are considered "cost-effective" [26].

We calculated the cost-effectiveness of the different screening strategies in two different ways. Firstly, we calculated the CER for each strategy as cost per life year saved in relation to no intervention. This measure of cost-effectiveness does not assume that all test technologies are equally standardisable, reliable over the long term, or acceptable. Secondly, we assessed cost-effectiveness if all strategies are considered equally feasible, and in this case calculated the Incremental CER (ICER) in relation to the next most cost-effective strategy. The first methodology reflects the policy-maker decision framework in the current setting, which currently has no widespread organised screening initiatives. Furthermore, it allows the analysis to independently consider the cost-effectiveness of each technology, which is potentially more appropriate in the current evaluation, since the standard cost-effectiveness analysis methodology can not take into account issues of stakeholder acceptability, test reliability or potential change or degradation in test performance over time for all the test methodologies included in the incremental cost-effectiveness analysis.

### Sensitivity analysis and supplementary calculations

Univariate sensitivity analysis was performed to assess the effect of model assumptions on the study findings, with the range of values used for sensitivity analysis given in Table 1. The range was chosen to reflect clinically feasible ranges and/or to encompass a wide range of possibilities. The primary analysis for the current study adopted a population perspective which takes achievable levels of screening participation into account. For comparison of the findings with a prior cost-effectiveness study on screening in other lower resource settings [27], we also calculated the reduction in lifetime risk of cancer in screened women only. We also assessed the impact of assuming perfect colposcopic accuracy for all health states, perfect sensitivity of the screening and diagnostic processes for detection of invasive cancer, no loss to follow-up in the diagnostic process, and no unsatisfactory test results. For the secondary analysis of cost-effectiveness using QALYs, we tested the effect of varying utilities within the range reported in the international literature.

We also performed a supplementary analysis to test the effect of assumptions about the natural history of HPV infection in women over 35 years of age on the calculated efficacy of once-lifetime *care*HPV screening. In the baseline model, prevalent infections observed in older women were assumed to be a mix of new infections and persisting infections; with the relative proportion of these at particular ages determined by the predictions of the dynamic transmission model (which was informed by reported female sexual behaviour in this population, as previously described). In this model implementation, it was assumed that all infections in older women had an equal probability of progression to CIN 3 and thus to invasive cancer. However, it is not known to what degree the HPV infections observed in women over 35 years in this population represent new infections acquired via new female or male partner contacts, or whether some (or most) of the prevalent infections represent persisting or previously latent but re-emerging infections, or to what degree cohort effects are evident in the relatively high rates of infection observed in the cross-sectional data for older women. Because of these uncertainties, the significance and potential for progression of a prevalent HPV infection in older women in this population is not well understood. Therefore, in a supplementary analysis we calculated the average reduction in cervical cancer lifetime incidence and mortality under the extreme assumption that all infections observed in women aged 35 years or older were persistent and had been originally acquired at younger ages, and were thus potentially detectable at a screening event at age 35 (this assumption would imply the highest possible efficacy for once-lifetime primary HPV screening at this age).

## Results

### HPV prevalence and cancer incidence and mortality, in the absence of intervention

**Figure 2:**
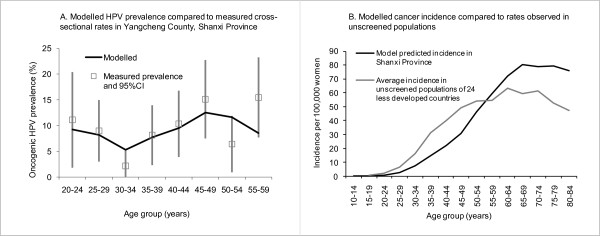
**Predicted and observed HPV prevalence and cervical cancer incidence**. Data on HPV prevalence in cytologically / histologically normal women (Figure 2A) obtained from analysis of Hybrid Capture II testing performed as part of the IARC/CICAMS study to obtain information on HPV positivity rates by 5-year age group [5]. The average incidence observed in unscreened populations of 24 less developed countries (Figure 2B) was calculated using data from IARC's Cancer Incidence in Five Continents Vol. VIII, which uses the Segi world standard population [14,15].

Figure 2A shows the measured and modelled age-specific oncogenic HPV prevalence in Shanxi Province (the HPV types implicated in cancer have been defined as oncogenic or "high risk" by the IARC [28]). The measured prevalence (using the HC2 test, which detects 13 HPV types, including HPV-16, -18, -31, -33, -35, -39, -45, -51, -52, -56, -58, -59, and -68) shows a "flat" or potentially "double-peaked" pattern, with relatively low levels of oncogenic HPV infection compared to developed countries but with relatively high rates in women over 40 years of age compared to younger women [5]. The measured age-standardised rate of infection for cytologically normal women aged 15-59 years was 8.1% (95%CI: 2.1% - 13.6%), and the model-fitted age-standardised prevalence was 8.2%.

We used a dynamic model of sexual behaviour and HPV transmission to estimate HPV incidence by single year of age, taking into account previously reported assumptions about the duration of infection and of naturally-acquired immunity [8,9]. The incorporation of the HPV incidence into the model of CIN natural history resulted in a predicted age-standardised incidence of invasive cervical cancer of 19.0 per 100,000 women, and a cumulative lifetime risk of cervical cancer of 2.9% (to age 85 years). These predictions are similar to the average age-standardised incidence of 22 per 100,000 (using the Segi world standard population), and the average cumulative lifetime incidence of 2.7%, observed in developing countries without substantial levels of screening calculated using data from IARC's Cancer Incidence in Five Continents Vol. VIII [14,15]) (Figure 2B). However, the age-specific patterns of invasive cancer in this population are predicted to differ somewhat from that in other populations, with the lower HPV infection rates in younger women resulting in lower rates of invasive cancer in women under 60 years of age (Figure 2B). The model also predicts an age-standardised cervical cancer mortality rate of 9.9 per 100,000 in the absence of any intervention; and a mortality to incidence ratio of 0.52 in this rural population, which is similar but slightly higher than the estimated overall ratio for China of 0.44 [29].

### Cancer incidence and mortality outcomes after screening interventions

Table 3 shows the predicted reductions in cervical cancer incidence and mortality and the cumulative lifetime risk of developing invasive cervical cancer to age 65 years. For all screening strategies assessed, the relative ordering of test technologies in terms of reduction in cervical cancer incidence and mortality was VIA-only (least effective); VIA/VILI; *care*HPV@1.0 pg/ml threshold; and *care*HPV@0.5 pg/ml (most effective). Once-lifetime screening strategies are predicted to reduce cancer incidence and mortality in the overall population by between 7-12% (depending on the test technology), with this reduction increasing to 13-24% for twice-lifetime screening strategies or to 17-54% for regular screening strategies. The reductions in mortality are predicted to be slightly higher than those for incidence, due to the effect of screening on detection of asymptomatic invasive cancer and some consequent downstaging. Comparable reductions are predicted in the cumulative lifetime incidence to age 65 years (Table 3).

**Table 3:** Predicted effect of screening strategies on cancer rates and cost-effectiveness


**Screening strategy and test**	**Average lifetime reduction cancer incidence**	**Average lifetime reduction cancer mortality**	**Average reduction cumulative incidence 0-64 years**	**Cost-effectiveness ratio compared to no intervention (US$ / LYS) †**

Once-lifetime (35 years)				
VIA only	7%	8%	8%	557
VIA/VILI	8%	10%	10%	629
*care*HPV@1.0 pg/ml	10%	12%	12%	959
*care*HPV@0.5 pg/ml	10%	12%	13%	909
Twice-lifetime (35+45 years)				
VIA only	13%	16%	17%	611
VIA/VILI	16%	18%	20%	689
*care*HPV@1.0 pg/ml	19%	22%	24%	1,032
*care*HPV@0.5 pg/ml	21%	24%	25%	985
10 yearly (30-59 years)				
VIA only	17%	19%	20%	665
VIA/VILI	19%	22%	23%	744
*care*HPV@1.0 pg/ml	24%	28%	29%	1,074
*care*HPV@0.5 pg/ml	24%	28%	29%	1,071
5 yearly (30-59 years)				
VIA only	27%	32%	33%	796
VIA/VILI	31%	36%	37%	916
*care*HPV@1.0 pg/ml	37%	43%	44%	1,395
*care*HPV@0.5 pg/ml	37%	43%	44%	1,391
IARC recommended				
VIA only	37%	43%	41%	1,213
VIA/VILI	41%	47%	45%	1,427
*care*HPV@1.0 pg/ml	47%	54%	52%	2,269
*care*HPV@0.5 pg/ml	48%	54%	52%	2,263

† Comparison GDP per capita Shanxi Province 2008: US$ 2,975 [25].

### Resource utilisation and optimal age range for once-lifetime screening

**Figure 3:**
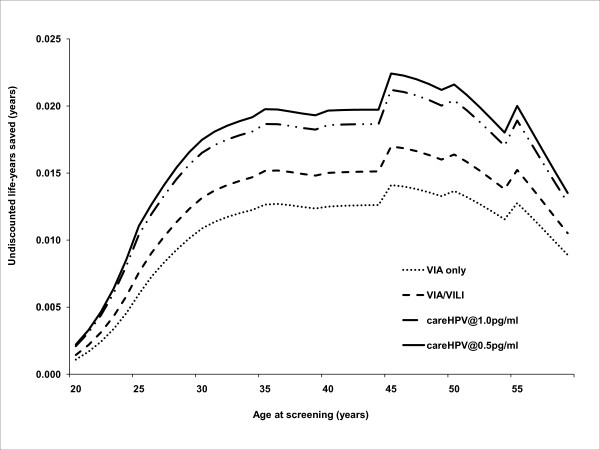
Analysis of most effective age of once-lifetime screening, showing life years saved (undiscounted) as a function of age at screening.

In order to assess differences in colposcopy referral and treatments performed for each of the screening tests, we calculated the predicted number of colposcopies, biopsies and treatments for once-lifetime screening. Table 4 details the numbers of each procedure performed after screening 100,000 women with each screening test at age 35 years. The number of colposcopies performed is related to the specificity of the screening test, and in this setting we found that approximately 6% of screened women would be referred to colposcopy after VIA screening, compared to approximately 8% of women after self-sampling with *care*HPV testing (Table 4). The predicted number of biopsies and treatments takes into account the mix of underlying natural histories referred to colposcopy by the screening test, the accuracy of colposcopy for each natural history state, and compliance with follow-up for additional visits. The colposcopy/treatment ratio, when considered in conjunction with the lifetime effects on cancer incidence and mortality, provides a measure of the efficiency of the screening and diagnostic process. *Care*HPV testing results in a colposcopy/treatment ratio of approximately 5 for either test threshold, whereas this is increased by 50% for VIA only (7.7) and over 300% for VIA/VILI testing (17.3), showing that fewer women are receiving unnecessary colposcopy in the case of *care*HPV. For once-lifetime screening strategies, maximum effectiveness in terms of life years saved for all screening tests was achieved if screening was performed between 35-50 years, with attenuated benefits in screening at ages over about 50-55 years (Figure 3).

**Table 4:** Predicted number of procedures per 100,000 women screened at age 35 years


**Screening test**	**Colposcopy**	**Biopsy**†	**Treatment for precancerous lesions**	**Colposcopy/ treatment ratio **

VIA only	6,068	1,920	791	7.7
VIA/VILI	17,881	4,640	1,036	17.3
*care*HPV@1.0 pg/ml	8,124	3,010	1,493	5.4
*care*HPV@0.5 pg/ml	8,478	3,164	1,587	5.3

† Biopsy does not include diagnoses from ECC.

### Cost-effectiveness outcomes

**Figure 4:**
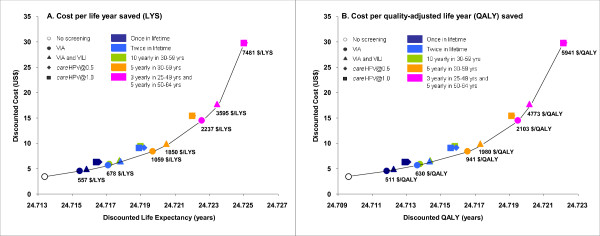
**Cost-effectiveness frontier for screening strategies**.

**Figure 5:**
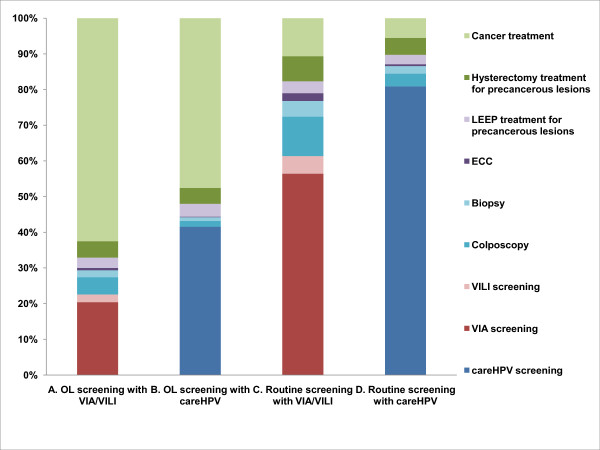
**The proportional breakdown of costs of screening and treatment for selected strategies**. A. Once-lifetime screening with VIA/VILI at age 35 years; B. Once-lifetime screening with careHPV@0.5 pg/ml at age 35 years; C. Routine screening with VIA/VILI at IARC-recommended intervals; D. Routine screening with careHPV@0.5 pg/ml at IARC-recommended intervals. The greatest component of the total lifetime cost is the cancer treatment cost for once-lifetime screening strategies. In contrast, for screening according to the IARC-recommended intervals (the most intensive regular screening strategy examined) the screening test costs dominate.

When compared to no intervention, all strategies would be considered cost-effective, because the cost per life year saved (CER) in relation to no intervention is in all cases considerably lower than the GDP per capita for Shanxi Province in 2008 (Table 3). Figure 4A provides a representation of the incremental cost-effectiveness of the strategies, showing the cost-effectiveness plane for costs versus life years and the relationship between screening technology, screening frequency and the relative increases in costs and effects. Using this method for assessing cost-effectiveness, strategies on the frontier (bottom right) of the cost-effectiveness plane are considered to be cost-effective. In this evaluation the visual inspection strategies that we assessed were generally found to be cost-effective (assuming consistent test performance over time), with VIA strategies at higher screening frequencies tending to dominate *care*HPV strategies at lower frequencies; this was driven by the low cost of visual inspection testing. As a further supplementary outcome we also calculated the ICER relative to the next most effective strategy using cost per QALYs (Figure 4B). The relative ordering of the strategies was generally unchanged when QALYs were considered; again, the visual inspection strategies at higher frequencies tended to dominate the *care*HPV strategies, assuming consistent test performance over time. We also assessed the proportional breakdown of costs as a percentage of the total lifetime cost of screening and treatment for various screening strategies, and these are presented in Figure 5.

### Sensitivity analysis and supplementary calculations

**Figure 6:**
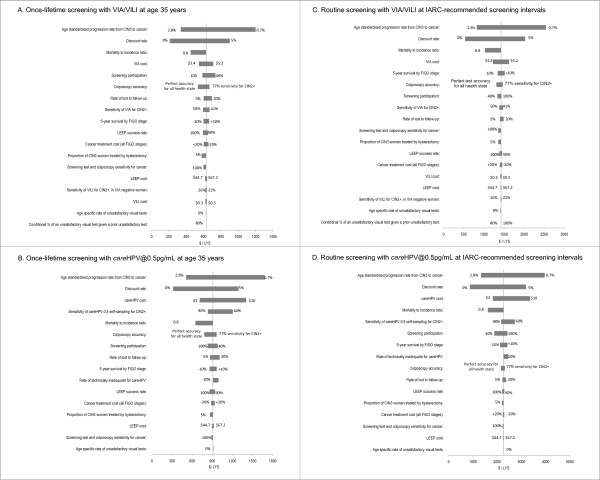
**Sensitivity analysis for cost per life year saved (LYS)**. Sensitivity analysis findings are shown for cost-effectiveness ratios relative to no intervention for selected test technologies (VIA/VILI and *care*HPV@0.5 pg/ml) and screening strategies (once-lifetime and IARC-recommended intervals); similar results were obtained for other screening strategies. In general, the cost-effectiveness findings were most sensitive to assumptions about the progression rate from CIN3 to invasive cervical cancer, the discount rate, mortality to incidence ratio, the cost of *care*HPV testing and VIA, test accuracy and cancer treatment costs. The baseline values and range for sensitivity analysis are provided in Table 1.

**Figure 7:**
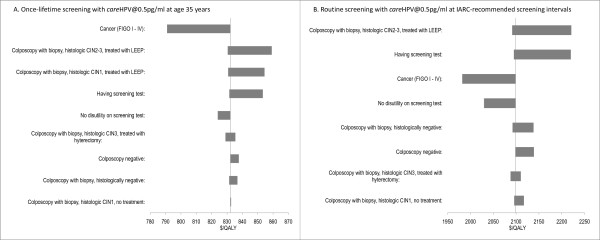
**Utilities sensitivity analysis for cost per quality-adjusted life years (QALY) saved**. Sensitivity analysis findings are shown for cost-effectiveness ratios calculated as cost per QALY saved relative to no intervention for *care*HPV testing at 0.5 pg/ml (once-lifetime screening and IARC-recommended intervals); similar relative results were obtained for other screening technologies. The baseline values and range for sensitivity analysis are provided in Table 1. Because this is a secondary sensitivity analysis, we examined two selected strategies as examples for QALY sensitivity analysis rather than the full four strategies considered in YLS analysis. Because the cost-effectiveness frontier curves calculated using YLS and QALYs in Figure 4A and 4B were similar, we focused the secondary sensitivity analysis on health state utility parameters.

For all screening strategies, the cost-effectiveness findings were most sensitive to assumptions about the progression rate from CIN3 to invasive cervical cancer, the discount rate, cancer survival assumptions (which affect the predicted mortality to incidence ratio), the cost of *care*HPV testing and VIA, test accuracy, and cancer treatment costs. The results of the sensitivity analyses are detailed in Figure 6 (for LYS outcomes) and Figure 7 (for QALY outcomes). The sensitivity analysis included consideration of a wide range of test characteristics for *care*HPV testing, in order to assess the impact of uncertainty about the accuracy of the test when translated to large-scale field implementation. If the sensitivity of *care*HPV testing for detection of CIN2+ were reduced to 60%, the estimated reduction in cancer incidence and mortality would decrease to between 8-45% (depending on the screening frequency).

In order to compare our findings for once-lifetime screening with those of a previous analysis in five developing countries (not including China) [27], we calculated the reduction in lifetime risk of invasive cervical cancer (to age 65 years) under two additional sets of assumptions. Firstly, we assumed lifetime risk was calculated in screened women only rather than taking a population perspective as for the current analysis. This increased the relative reduction in lifetime risk of invasive cervical cancer to between 8-13% (depending on the test technology in screened women). Secondly, we adopted other key assumptions of the prior analysis, including see-and-treat procedures for visual inspection and HPV screening strategies, an assumed sensitivity for CIN2+ for colposcopy, VIA and HPV testing of 100%, 76% and 88%, respectively; and no unsatisfactory visual tests. Under these assumptions the predicted reduction in lifetime incidence after a single screening test at age 35 years in screened women was between 21% (for VIA) and 24% (for *care*HPV testing). This is broadly consistent with the result of the prior analysis which found that once-lifetime testing at age 35 years would be associated with a reduction of between 25-36% in lifetime risk to age 65 years in screened women [27]. The differences in the assumed sensitivity of the screening tests, the perspective taken (screened individual vs population perspective) and to some extent the requirement for colposcopic confirmation in the current context explained most of the differences between our study findings and that of the prior analysis.

Under the extreme assumption that all infections observed in this population at age 35 years or older were persisting and were originally acquired at a younger age (the assumption most favourable to once-lifetime *care*HPV screening at age 35 years), the average lifetime reductions in cancer incidence and mortality after once-lifetime *care*HPV screening at 0.5 pg/ml are predicted to increase to 26% and 27%, respectively. Therefore, under these differing assumptions about the natural history of HPV in older women, the predicted lifetime reductions in cancer rates associated with once-lifetime screening with *care*HPV are predicted to increase by more than a factor of two.

## Discussion

To our knowledge, this is the most detailed modelled evaluation of cervical cancer screening to be conducted in China. The analysis incorporated setting-specific information on the risks of HPV infection, screening and management procedures and the costs associated with screening, diagnosis and treatment. The screening alternatives considered were based on extensive consultation with local key opinion leaders about the practical options for organising screening in rural China. We evaluated the predicted outcomes resulting from various screening alternatives, including once or twice-lifetime screening at ages 35 and/or 45 years by mobile screening units; and routine screening at 5-yearly, 10-yearly and IARC-recommended intervals within a program of regular screening. Depending on the test technology used, and assuming a participation rate of ~70%, we found that once-lifetime screening at age 35 years would reduce age-standardised cervical cancer mortality in the population by 8-12% over the long term, with a CER of $557-959 per LYS. Regular screening at a feasible age-standardised participation rate of 62% in women aged 30-59 years would reduce cervical cancer mortality by 19-54%, with a CER of $665-2,269 per LYS. Although these findings imply that all strategies would be considered cost-effective in relation to no intervention, testing with *care*HPV is predicted to lead to greater absolute benefits when compared to visual inspection tests.

The screening participation rate used in the evaluation was informed by a government-sponsored VIA/VILI screening demonstration project. Although the assumed participation rate may seem relatively high in context of developed country programs, it has been demonstrated to be achievable locally, in the context of a high level of population enumeration and high compliance with government initiatives. We found that in this population in rural China a single round of screening can potentially be extended from 35 year old women to women aged up to 50 years without loss of effectiveness. This finding is important for the practical realisation of single round screening strategies as it implies that a reasonably wide age range of women can be included whilst still maximising long term benefits. However, we found that the lifetime benefits of once-off screening may be lower than previously estimated for other populations [27]. A previous study of the cost-effectiveness of cervical screening in five developing countries (not including China) concluded that once-lifetime screening at age 35 years could reduce the cumulative lifetime risk of invasive cervical cancer in screened women by between 25-36%, depending on the screening strategy and technology used [27]. After taking into account a population rather than an individual perspective, local information on the sensitivity of screening tests, the local requirement for colposcopic confirmation of all screening test results with consequent diagnostic accuracy loss, and other factors, our analysis for rural China identified more attenuated benefits. Our findings are complementary to that of a recent randomised trial in rural India which found that a single round of HPV screening was associated with a reduction by approximately 50% in the hazard ratio for the development of advanced cervical cancer and cervical cancer death over the following eight years [30]. Over a lifetime, the relative protective effect of a single HPV test in screened versus unscreened women is expected to be reduced as some new HPV infections and new cancer cases accrue in both groups over time. Our study supplements the trial findings by providing a measure of the benefits of a single round of screening but considered from the standpoint of long term (lifetime) follow-up.

Our finding that *care*HPV screening can be cost-effective in rural China is consistent with that of a prior analysis by Levin et al [31]. However, we focused on the differential outcomes and cost-effectiveness associated with visual inspection methods and *care*HPV testing, considering the differential effects associated with varying the HPV test threshold or adjusting the visual methodology to include Lugol's iodine. In contrast, prior work considered *care*HPV testing in relation to HC2 testing and cervical cytology, which may not be readily practicable alternatives in this setting. Therefore, the current paper extends the findings of the related previous work [27,31] in two important ways - it provides more detail for this particular setting, and it compares the performance of *care*HPV screening to the other currently viable test alternatives in this setting.

Visual inspection methods have been proposed as low cost screening solutions in less developed countries [7,30,32]. However, problems with accuracy and test standardisation may have hindered the widespread adoption of visual testing modalities, and a round of VIA screening was not associated with any significant reduction in the risk of advanced cancer or death over eight years of follow-up in a recent large scale trial in India [30]. It has been suggested that in some diagnostic accuracy studies of visual inspection methods the sensitivity of the test may have been overestimated by up to 20 absolute percentage points due to verification bias when colposcopically-directed biopsy is used as the gold standard [17]. In order to increase the sensitivity of visual testing, the addition of VILI has been proposed as a secondary test for VIA-negative women [7]. The VIA/VILI combined procedure may moderately increase the sensitivity of visual inspection modalities [32] and has been shown to be logistically feasible within a government-sponsored large-scale demonstration project at 42 sites across China, which was rolled out from 2006 [18]. However, limited data are available on the accuracy of VIA/VILI as a combined procedure. Based on re-analysis of follow-up data from a previous study [5,16], we assumed that in this setting the addition of VILI would increase sensitivity but would decrease the overall specificity of visual inspection testing, which may not uniformly be the case [32]. Therefore, more data on the accuracy of combined VIA/VILI testing is required, and our results for this combined test modality should be considered provisional.

This analysis has several limitations. Firstly, the model which was used is based upon CIN natural history states rather than states that directly reflect type-specific HPV infection persistence and progression. It should be noted that there are considerable uncertainties about the natural history of HPV (and thus modelled analysis of primary HPV testing) in populations such as the one we studied in rural China, in which the rates of infections at older ages are comparable or higher to those at younger ages. The relative proportion of new versus re-emerging latent or persisting HPV infections in women over 35 years of age in this population is not known. Furthermore, the progressive potential of new infections in older women in this population is difficult to characterise. We did not have direct information on male sexual behaviour and thus could not estimate the total rate of new exposures in older women; therefore we took a fitting approach to modelling the observed pattern of female HPV infection by incorporating survey data on female behaviour and estimating male behaviour patterns. In general terms, if infections in women aged 35 years or older have a low potential to progress to CIN3 (and then to invasive cancer), then the efficacy of once-lifetime HPV screening at age 35 would be higher. In our baseline analysis we assumed that some new infections would progress to CIN3 after a once-lifetime screening event. However, under the extreme assumption that all HPV infections occurring in older women that have the potential to progress to CIN 3 are potentially detectable (and treatable) at the time of the screening event, the average reduction in cumulative lifetime incidence and mortality associated with once-lifetime HPV screening would increase. This implies that from the perspective of the cost-effectiveness of once-lifetime primary HPV screening, our evaluation has taken a conservative approach.

Another limitation of the study is that we could not account for variation in screening and diagnostic test performance over time, due to drift in clinical or laboratory practice (although we did include consideration of the need for quality control procedures in the costs associated with the screening strategies). Because of this limitation, we presented our main cost-effectiveness findings for each technology as a cost-effectiveness ratio compared to no intervention, since the relative test performance of visual inspection and HPV testing methodologies may vary over time in this setting in as-yet-unpredictable ways. Another limitation of the study is that we did not have extensive local data on health state preferences (utilities) or data from comparable populations to use in the calculation of QALYs, and therefore, the primary findings of the current analysis were based on the estimation of life years saved for the various screening strategies.

We have extensively reviewed the available local cervical cancer incidence and mortality data. Shanxi Province has been thought to be a "high risk" area for cervical cancer; but in practice the evidence for this appears to depend heavily on the findings of the earliest cancer mortality surveys conducted in China in the 1970s which found that rates were up to 83 per 100,000 women (according to the Segi World Standard Population [2,33]; no information on the population according to Ahmad et al can be calculated because age-specific data are not available [14]). Two subsequent mortality surveys were performed in China [2,34,35] - the most recent in 2000-2005 included data from Xiangyuan county in Shanxi Province, finding a much lower mortality rate of 6.8 per 100,000 women [2,35] (according to the Chinese standard population of 1982; again, age-specific data are not available to calculate rates according to the Ahmad et al standard population). Furthermore, perceptions of the region as "high risk" appear inconsistent with the relatively low rates of HPV infection observed in females overall (Figure 2A in our paper). There are no IARC-certified cancer registries in the region and so no up-to-date accurate registry information is available. In the absence of such information we have taken a conservative approach to the current evaluation, and assumed the lifetime risk of cancer and the average incidence rate are equivalent to the average rate of developing countries. It should be noted that this incidence rate is still much higher than the rate reported for China overall according to the most recent data from IARC's Cancer Incidence in Five Continents [36], which is less than 4 per 100,000 women and substantially less than many developed countries. If in fact cancer rates are higher than we assumed in this setting, this would have the effect of increasing the estimated cost-effectiveness of all screening strategies, but would be unlikely to change the relative costs and benefits of the various strategies, compared to no intervention.

Our objective in this study was to provide policy makers in China with the best available evidence on the relative benefits and costs of different screening strategies. Modelling is a commonly used approach, and here we have employed the techniques of modelling to integrate local evidence with international data. Modelling is an important complement to randomised clinical trials, but one of the advantages of modelling studies is that they can predict lifetime epidemiologic outcome and cost-effectiveness results. Additionally, a much greater range of potential strategies can be evaluated via modelled approaches. A total of 20 screening strategies were evaluated in our study, but such a large number of strategies are unlikely to be practical in a large-scale screening trial. Although randomised controlled trials are ultimately the "gold standard", in practice these are highly unlikely to be performed in every setting into which cervical cancer prevention initiatives are introduced. In the absence of such locally conducted trials, modelling studies provide local policy makers with the best available information with which to inform further decision making.

Our experience in working with local key opinion leaders and policy makers in China emphasises the importance of performing detailed evaluations of the viable alternative strategies taken in the context of local practices, feasible methods of screening organisation, and clinically acceptable options; rather than generically applying strategies that may be applicable in other settings. For example, although visual inspection with cryotherapy see-and-treat has previously been evaluated as cost-effective in other settings [27], this strategy would not be acceptable in China. This is because power supplies for LEEP would be available in the settings we considered; and the in context of local clinical practice it is felt that the lack of histological diagnosis after cryotherapy would potentially increase the risk of medical disputes and confusion about clinical management. The local preference for diagnostic confirmation followed by LEEP treatment is emphasised by the incorporation of this management strategy into a recently implemented large scale government demonstration project for VIA/VILI screening which has the objective of screening up to 10 million women [37]. Our evaluation models the VIA/VILI management processes used in the demonstration project, but also gives local policy makers much more detailed information on the relative benefits of *care*HPV screening. In relation to the sampling method for HPV testing, we evaluated each method in context of the associated practically realisable method(s) of screening organisation, so that self-sampling (which is less costly than health provider sampling) was combined with community/village clinic-based once- or twice-lifetime screening. Health provider sampling was combined with county hospital-based regular screening, which would be viable in this more centralised approach. However, it should be noted that because the evidence suggests that both provider and self-sampling for *care*HPV testing has relatively high sensitivity [3], the effectiveness of the two sampling strategies are also expected to be similar.

The strengths of the current study lie in the detailed epidemiological modelling and the use of extensive local information on screening and diagnostic test accuracy and management practices, age-specific unsatisfactory rates, and costs. Our study takes into account measured patterns of sexual behaviour and HPV infection in this rural population. This has allowed us to characterise in detail the potential role of primary HPV screening in relation to other viable alternatives in this important low-resource setting.

## Conclusions

This modelled analysis suggests that primary HPV screening with *care*HPV would be an effective and cost-effective method of primary screening in rural China, and that it compares favorably to visual inspection methods in terms of increased effectiveness.

## List of abbreviations

CER: cost-effectiveness ratio; CICAMS: Cancer Institute of Chinese Academy of Medical Sciences; CIN: cervical intraepithelial neoplasia; CNY: Chinese Yuan; ECC: endocervical curettage; FIGO: International Federation of Gynecology and Obstetrics; GDP: gross domestic product; HPV: human papillomavirus; IARC: International Agency for Research on Cancer; ICER: incremental cost-effectiveness; LEEP: loop electrosurgical excision procedure; LYS: life year saved; QALYs: quality-adjusted life years saved; VIA: visual inspection with acetic acid; VILI: visual inspection with Lugol's iodine; WHO: World Health Organization.

## Competing interests

The authors declare that they have no competing interests.

## Authors' contributions

JFS, KC, JBL and YLQ had full access to all interim outputs and data from the model, and had responsibility for the integrity of the study and accuracy of the analysis. YLQ and KC were responsible for the study concept and design. JFS, YN, FHZ, LM, LS, YJK, YZZ, MAS and XXF acquired the data used for the model via field studies and literature review. JFS, KC, JBL and YLQ analysed and interpreted the data, and conceived the management pathways used. JFS and KC drafted the manuscript. RL, FHZ, JFC and YLQ revised the manuscript. JBL and KC were primarily responsible for model implementation with assistance from MAS. All authors read and approved the final manuscript.

## Pre-publication history

The pre-publication history for this paper can be accessed here:

http://www.biomedcentral.com/1471-2407/11/239/prepub
